# Author Correction: Climate resilience of European wine regions

**DOI:** 10.1038/s41467-026-73655-3

**Published:** 2026-05-25

**Authors:** Simon Tscholl, Sebastian Candiago, Thomas Marsoner, Helder Fraga, Carlo Giupponi, Lukas Egarter Vigl

**Affiliations:** 1 Institute for Alpine Environment, Eurac Research, Viale Druso 1, 39100 Bozen/Bolzano, Italy; 2 Department of Ecology, University of Innsbruck, Innrain 52, 6020 Innsbruck, Austria; 3 Department of Economics, Ca’ Foscari University of Venice, S. Giobbe 873, 30121 Venezia, Italy; 4 Professorship of Ecological Services, Bayreuth Center of Ecology and Environmental Research (BayCEER), University of Bayreuth, Universitätsstraße 30, 95447 Bayreuth, Germany; 5 Centre for the Research and Technology of Agro-Environmental and Biological Sciences (CITAB), Institute for Innovation, Capacity Building and Sustainability of Agri-food Production (Inov4Agro), Universidade de Trás-os-Montes e Alto Douro (UTAD), 5000-801 Vila Real, Portugal

**Keywords:** Governance, Agroecology, Climate-change ecology, Plant ecology

Correction to: *Nature Communications* 10.1038/s41467-024-50549-w, published online 24 July 2024

In the version of this article initially published, there was a typographical error in the Fig. 1c legend, now reading “Histogram showing the distribution of HI, CNI and DI for the periods 1981–2010 and 2071–2100 under the ssp370 scenario of all wine regions,” where 2071 appeared originally as “1971.” The legend has been amended in the HTML and PDF versions of the article.

