# Supplementary material for: Climate resilience of European wine regions
Source: Nat Commun. 2024 Jul 24;15:6254. doi: 10.1038/s41467-024-50549-w (PMC11269675; doi:10.1038/s41467-024-50549-w)
Supplement: Supplementary file 1 — Supplementary Information [file 41467_2024_50549_MOESM1_ESM.pdf]

Supplementary Information for “Climate resilience of European wine regions”

Simon Tscholl*[1,2], Sebastian Candiago*[1,3,4], Thomas Marsoner[1], Helder Fraga[5], Carlo Giupponi[3], Lukas Egarter Vigl[1]

1. Institute for Alpine Environment, Eurac Research, Viale Druso 1, 39100 Bozen/Bolzano, Italy
2. Department of Ecology, University of Innsbruck, Innrain 52, 6020 Innsbruck, Austria
3. Department of Economics, Ca' Foscari University of Venice, S. Giobbe 873, 30121 Venezia, Italy
4. Professorship of Ecological Services, Bayreuth Center of Ecology and Environmental Research (BayCEER), University of Bayreuth, Universitätsstraße 30, 95447 Bayreuth, Germany
5. Centre for the Research and Technology of Agro-Environmental and Biological Sciences (CITAB), Institute for Innovation, Capacity Building and Sustainability of Agri-food Production (Inov4Agro), Universidade de Trás-os-Montes e Alto Douro (UTAD), 5000-801 Vila Real, Portugal

corresponding author: Simon Tscholl (simon.tscholl@eurac.edu)
*These authors contributed equally

Contents

Supplementary Figures 2
Supplementary Note 1: Description and calculation of individual indicators 9
Indicators: Exposure and Sensitivity 9
Indicators: Adaptive capacity 10
Supplementary Note 2: Validation of the bioregional climate range 18
Supplementary References 20

Supplementary Figures

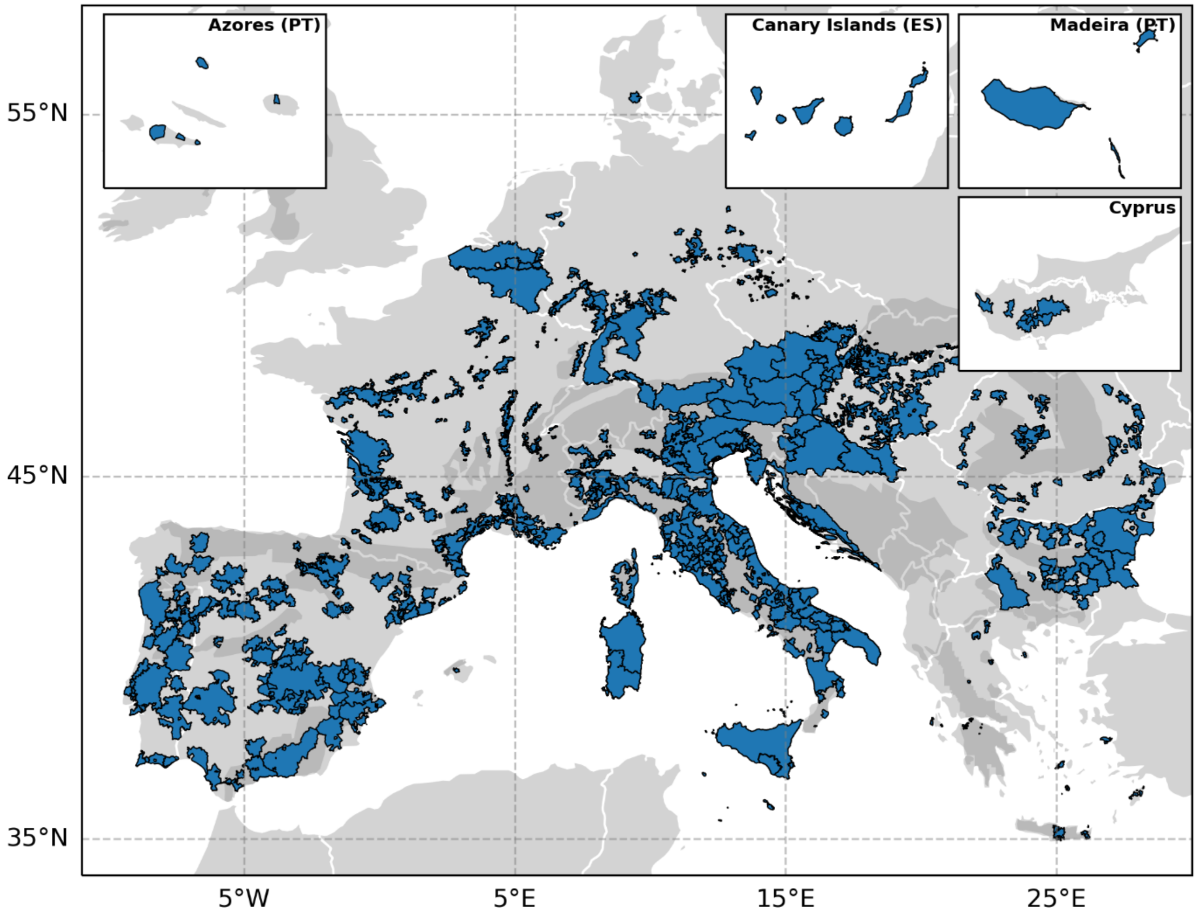

Supplementary Figure 1: European wine PDOs (n=1085). The dataset is described in Candiago, S., Tscholl, S., Bassani, L., Fraga, H. & Egarter Vigl, L. A geospatial inventory of regulatory information for wine protected designations of origin in Europe. Sci Data 9, 394 (2022) and was downloaded from Candiago, S., Tscholl, S., Bassani, L., Fraga, H. & Egarter Vigl, L. A geospatial inventory of regulatory information for wine Protected Designations of Origin in Europe. Figshare (2022) doi:10.6084/m9.figshare.c.5877659.v1. Dark grey areas refer to mountain regions. Made with Natural Earth. Free vector and raster map data @ naturalearthdata.com.

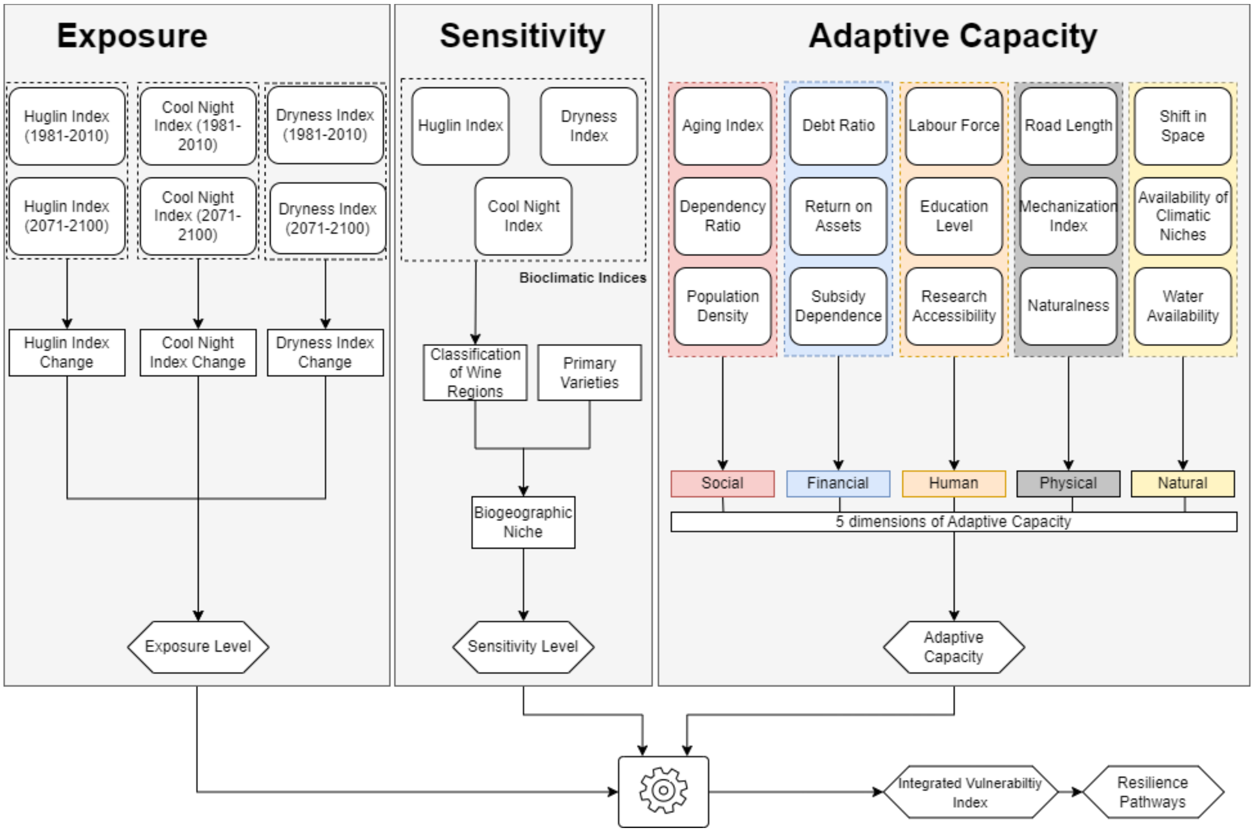

Supplementary Figure 2: Methodological overview. The three grey boxes show the steps and the indicators used to calculate exposure, sensitivity, and adaptive capacity.

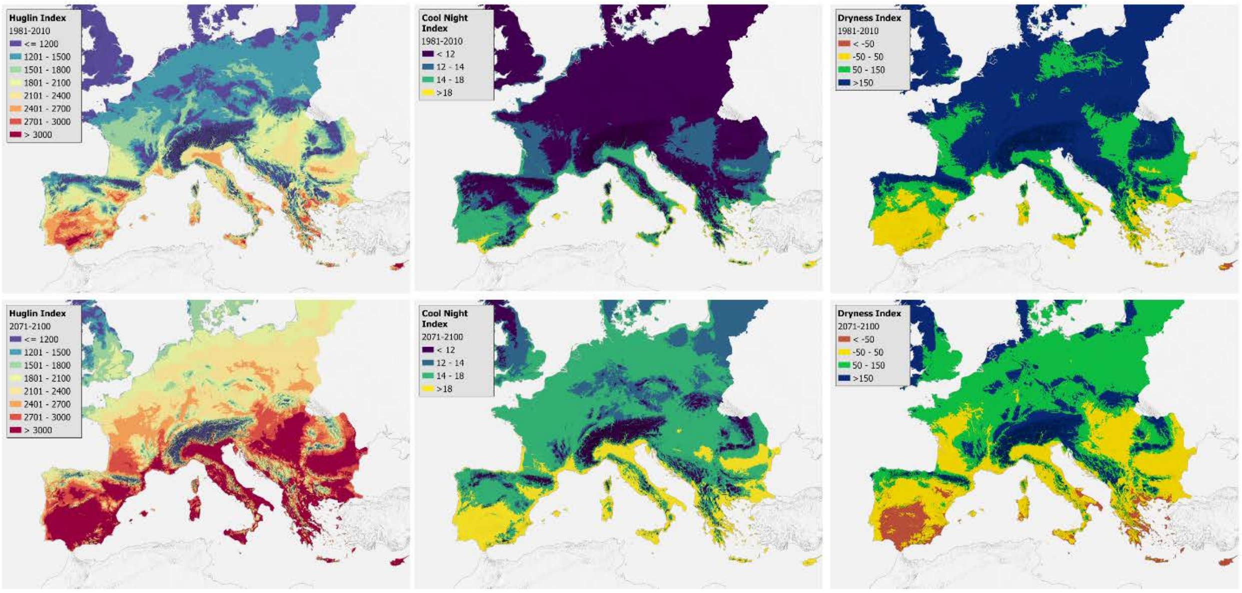

Supplementary Figure 3: The three bioclimatic indices (Huglin Index, Cool Night Index, Dryness Index) during two time periods. The upper row shows the index values during the period 1981-2010 and the bottom row during the period 2071-2100. Made with Natural Earth. Free vector and raster map data @ naturalearthdata.com.

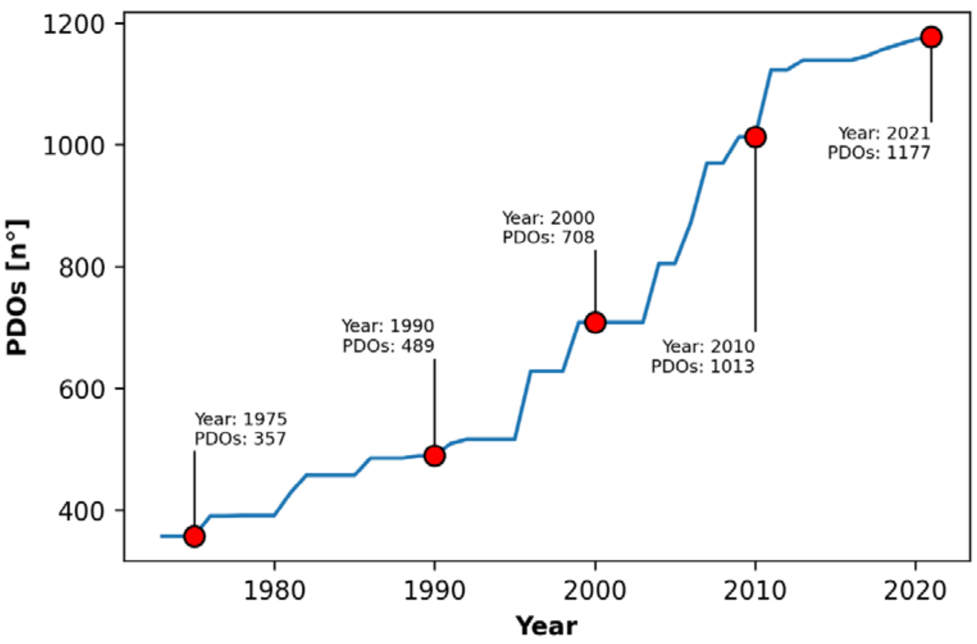

Supplementary Figure 4: Registered PDO regions in Europe per year. The red points highlight the number of registered PDO regions for selected years.

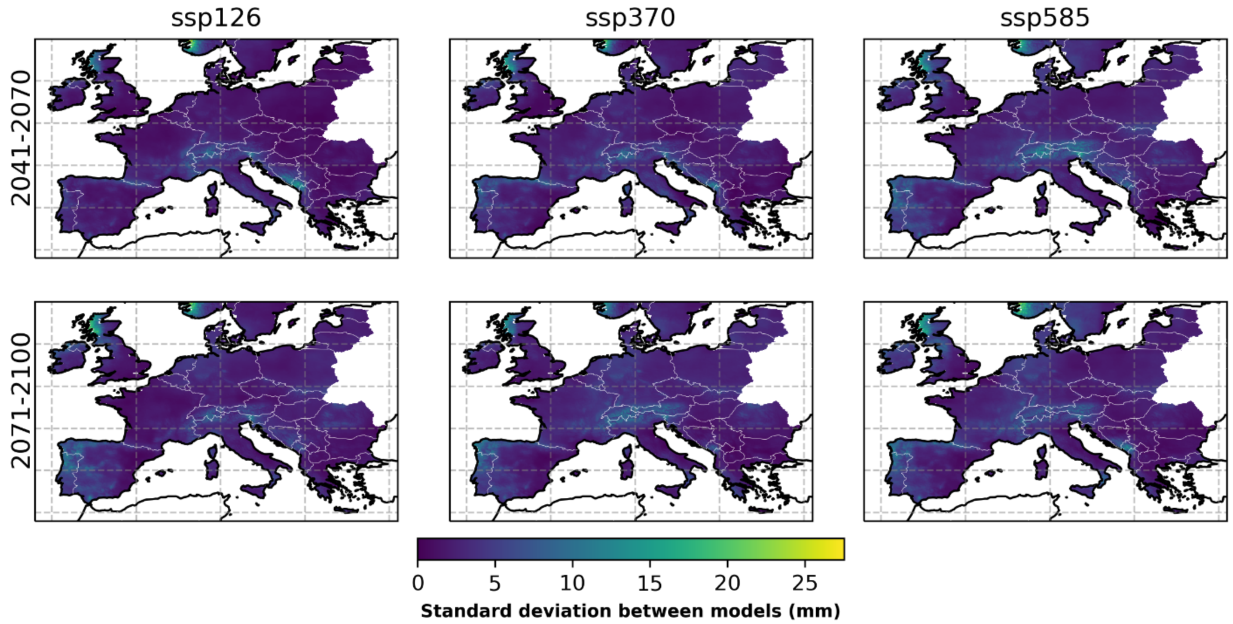

Supplementary Figure 5: Model spread for annual precipitation over 5 GCMs, shown for two periods and three scenarios. The colour indicates the standard deviation between the five models used in the present analysis for each pixel. Made with Natural Earth. Free vector and raster map data @ naturalearthdata.com.

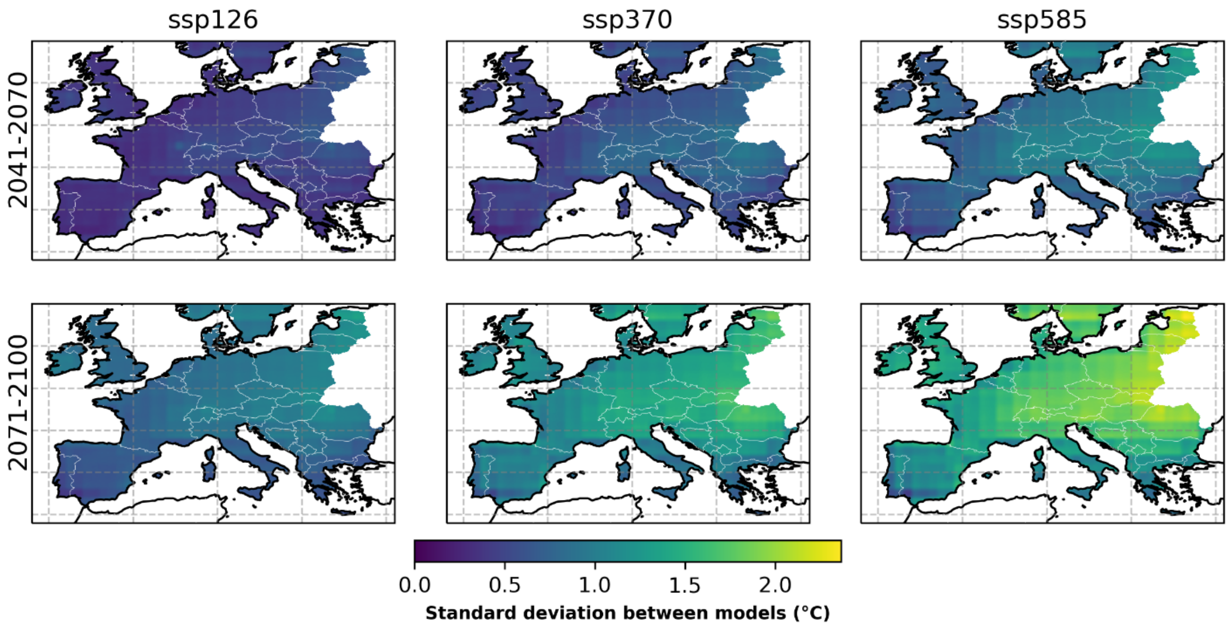

Supplementary Figure 6: Model spread for annual mean temperature over 5 GCMs, shown for two periods and three scenarios. The colour indicates the standard deviation between the five models used in the present analysis for each pixel. Made with Natural Earth. Free vector and raster map data @ naturalearthdata.com.

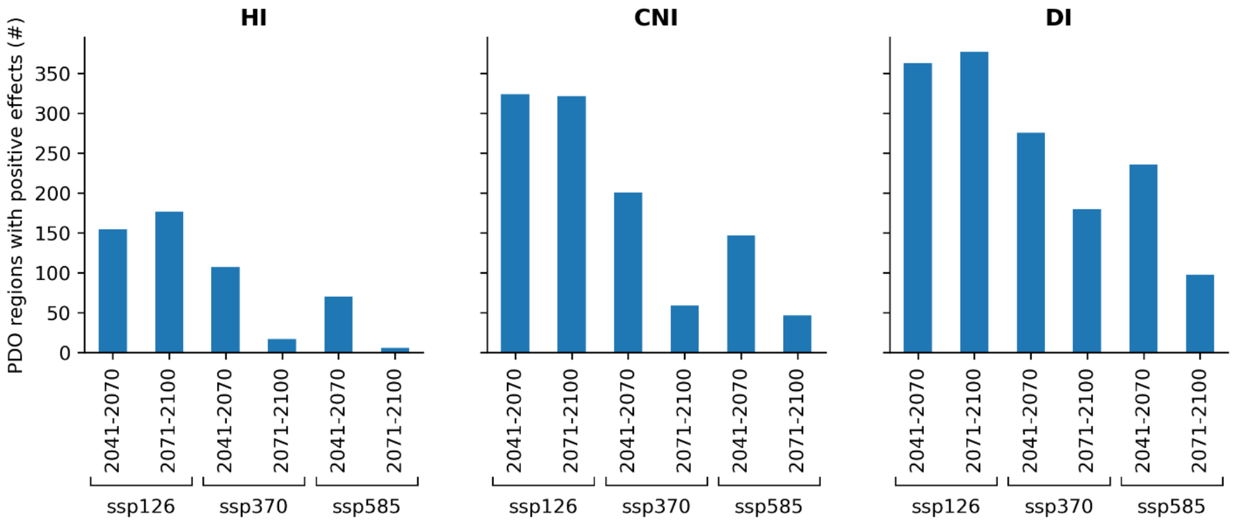

Supplementary Figure 7: Number of PDO regions authorizing varieties with potentially positive impacts from climate change based on three different bioclimatic indices. The number of regions was calculated based on all investigated PDO regions (n=1085) by extracting those regions with varieties that might benefit from climate change. The results are presented separately for each bioclimatic index, two time periods and three scenarios. HI = Huglin Index, DI = Dryness Index, CNI = Cool Night Index.

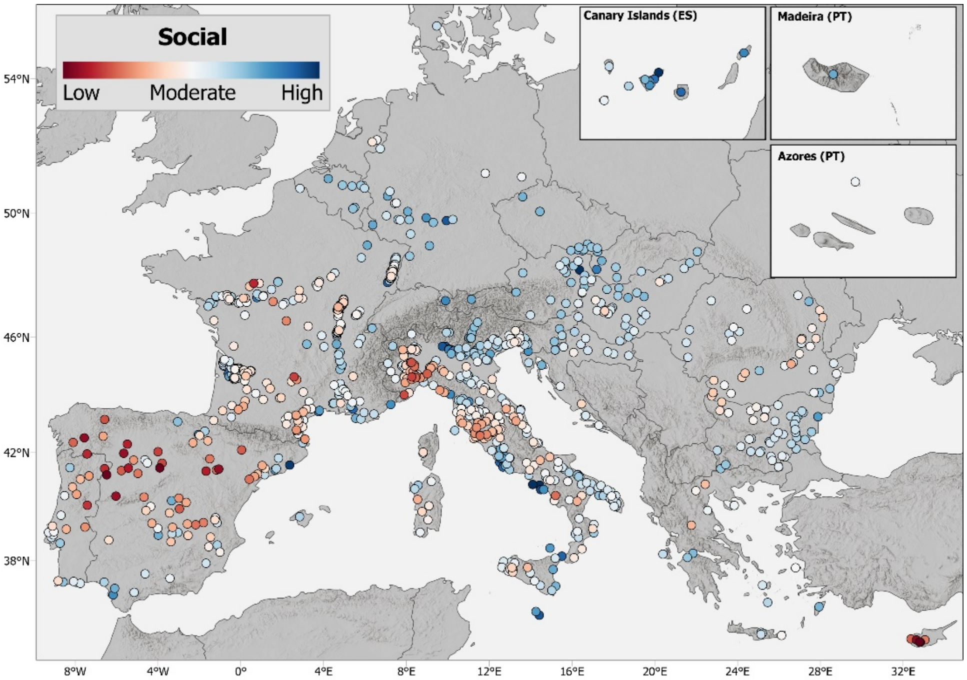

Supplementary Figure 8: The social dimension of the adaptive capacity. Values are based on the aggregation of the aging index, dependency ratio and population density indicators. The points refer to the centroids of the PDO regions (n=1085). Dark grey areas refer to mountain regions. Made with Natural Earth. Free vector and raster map data @ naturalearthdata.com.

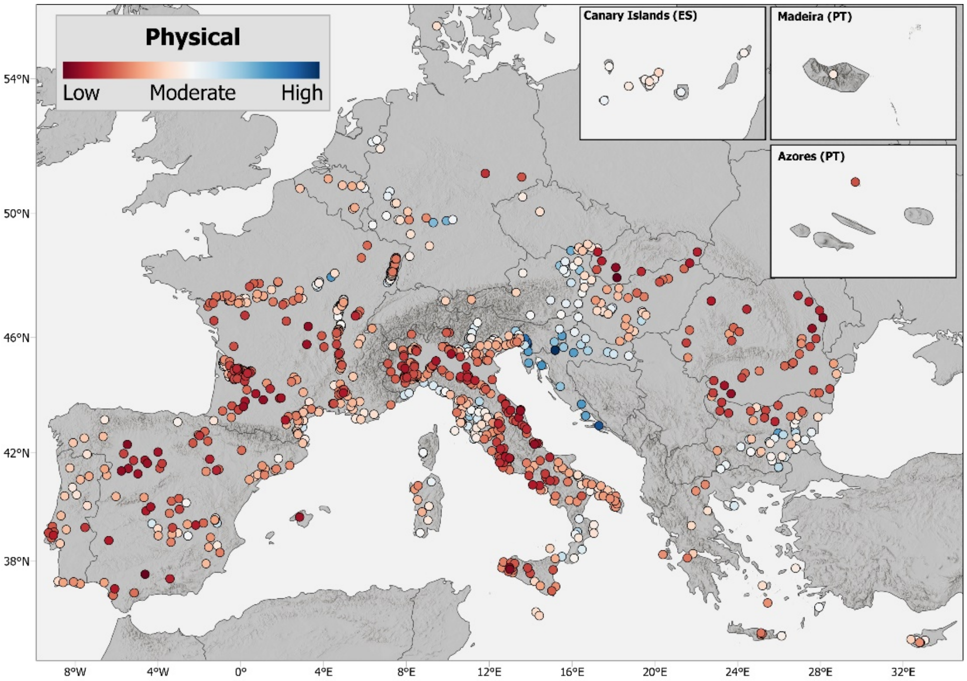

Supplementary Figure 9: The physical dimension of the adaptive capacity. Values are based on the aggregation of the road length, mechanization index and naturalness indicators. The points refer to the

centroids of the PDO regions (n=1085). Dark grey areas refer to mountain regions. Made with Natural Earth. Free vector and raster map data @ naturalearthdata.com.

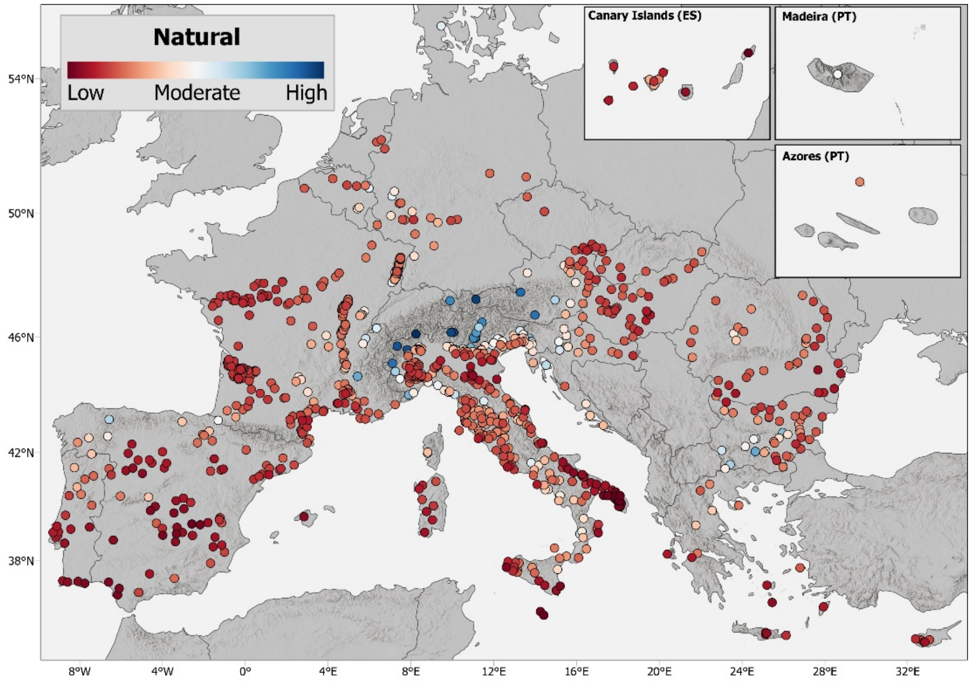

Supplementary Figure 10: The natural dimension of the adaptive capacity. Values are based on the aggregation of the shift in space, water availability and availability of climatic niches indicators. The points refer to the centroids of the PDO regions (n=1085). Dark grey areas refer to mountain regions. Made with Natural Earth. Free vector and raster map data @ naturalearthdata.com.

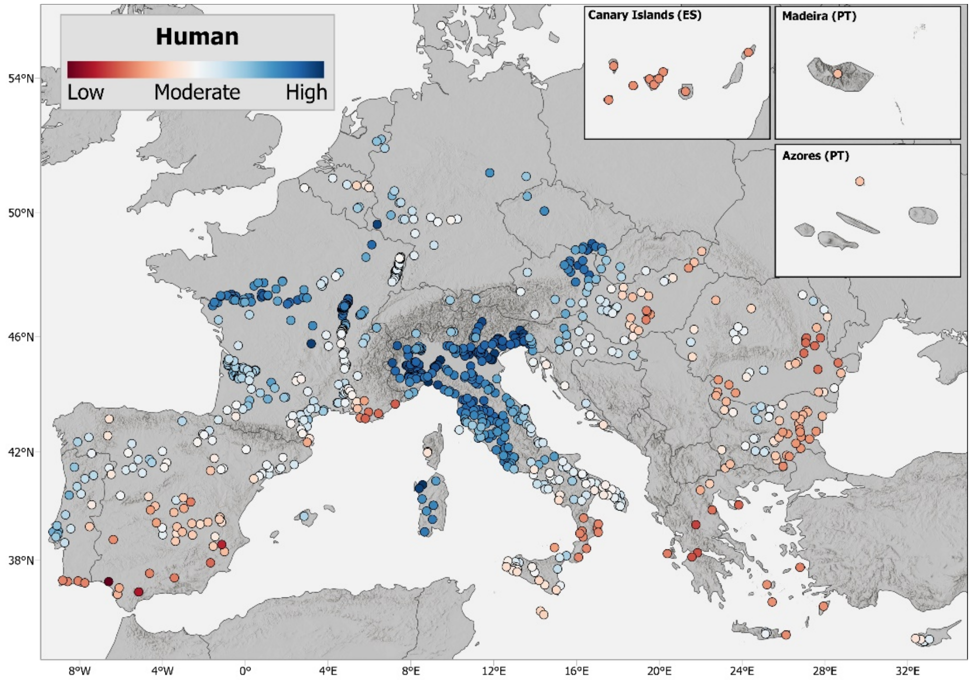

Supplementary Figure 11: The human dimension of the adaptive capacity. Values are based on the aggregation of the labour force, education level and research accessibility indicators. The points refer to the centroids of the PDO regions (n=1085). Dark grey areas refer to mountain regions. Made with Natural Earth. Free vector and raster map data @ naturalearthdata.com.

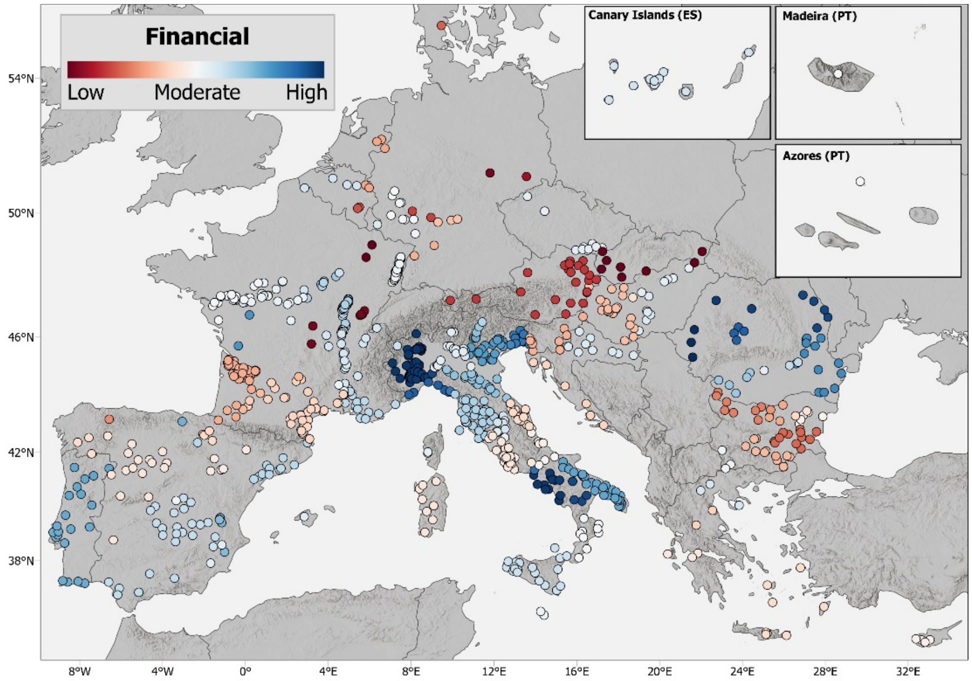

Supplementary Figure 12: The financial dimension of the adaptive capacity. Values are based on the aggregation of the debt ratio, return on assets and subsidy dependence indicators. The points refer to the centroids of the PDO regions (n=1085). Dark grey areas refer to mountain regions. Made with Natural Earth. Free vector and raster map data @ naturalearthdata.com.

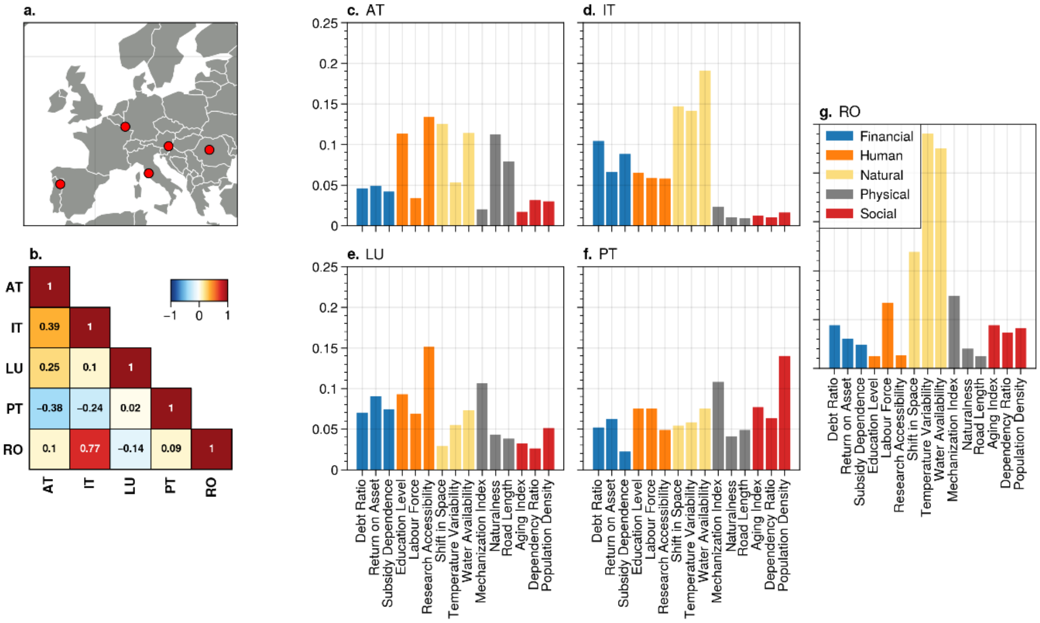

Supplementary Figure 13: Results from the survey on indicator importance of the adaptive capacity

indicators in five European PDO regions. a) The map shows the location of the five PDO regions for which the survey was carried out. Made with Natural Earth. Free vector and raster map data @ naturalearthdata.com. b) Pearson correlation between the weights of the individual regions. The colour of the squares indicates the strength of the correlation, from -1 to 1. c-g) The weights for all 15 indicators from the survey for each region. Each indicator is assigned a weight between 0 and 1 based on the results from the analytical hierarchy process. The bar colours indicate the different dimensions. AT = Südsteiermark, LU = Moselle Luxembourgeoise, IT = Maremma Toscana, PT = Douro, RO = Târnave.

Supplementary Note 1: Description and calculation of individual indicators

Indicators: Exposure and Sensitivity

Huglin Index

**General description**<br>The Huglin index describes the thermal conditions in a wine region during the vegetation period, which is related to the potential grape sugar content[1], (C°).
**Rationale**<br>Thermal conditions during the vegetation period play a critical role for vine phenology and grape ripening[2]. They strongly determine viticultural suitability[3] and have been used to forecast required adaptation efforts for several regions[4]. Changes in thermal conditions therefore have significant impacts on the wine style that can be produced in a region as well as which varieties can be cultivated and where they can be grown.
**Input data**<br>Chelsa high resolution monthly temperature data (1981-2010)[5,6].
**Calculation process**<br>$$\sum_{April}^{Sept.} \frac{(T-10)+(T_{max}-10)}{2} d$$<br>T = Mean air temperature (C°)<br>T_{max}= Maximum air temperature (C°)<br>d = Length of day coefficient, from 1.02 to 1.06
**Standardization**<br>0 = lower Huglin index; 1 = higher Huglin index.

Cool night index

**General description**<br>The cool night index accounts for minimum temperatures during September, providing an estimate of temperature conditions during the ripening stage[1], (C°).
**Rationale**<br>Minimum temperatures during grape ripening are critical for grape composition at harvest and influence wine style and quality. As a consequence, increased air temperature during this period can result in decreased wine quality[7]. For instance, if minimum air temperatures during night become too

high, sugar consumption in the berries increases which in turn affects the concentration of flavour compounds[7].
**Input data**<br>Chelsa high resolution monthly temperature data (1981- 2010)[5,6].
**Calculation process**<br>Average daily minimum temperature during September (C°).
**Standardization**<br>0 = lower cool night index; 1 = higher cool night index.

Dryness index

**General description**<br>The dryness index evaluates soil water availability for vine development, by estimating soil water reserves, precipitation, and potential evapotranspiration[1], (mm).
**Rationale**<br>The available water is a critical factor that influences vine growth and berry ripening. For instance, a decrease in precipitation or an increase in temperature in areas with dry conditions (low dryness index) can put the vines under water stress and negatively impact vine vigor[7]. In contrast, areas with moist conditions (high dryness index) might even experience positive consequences from a decrease in the dryness index, such as lower pathogen pressure[8].
**Input data**<br>Chelsa high resolution monthly temperature and precipitation data (1981- 2010)[5,6].
**Calculation process**<br>$$\sum_{April}^{Sept.} (W_0 + P - T_v - E_s)$$<br>Wo=Initial available soil water reserve (mm) on the first month / DI on the following months<br>P = Precipitation (mm)<br>T_v = Potential vineyard transpiration (mm)<br>E_s = Direct evaporation from the soil (mm)<br>T_v and E_s are assessed using the Thornthwaite method
**Standardization**<br>0 = higher dryness index; 1 = lower dryness index.

Indicators: Adaptive capacity

Aging index

**General description**<br>The ratio between the old and the young population in a municipality.
**Rationale**<br>This indicator is a measure of aging population indicating how many persons at retirement age there are for every child. It shows the demographic trend that a municipality can expect. An unfavourable ratio (high indicator values) may indicate an exodus of young people and families, showing a negative outlook for the regional labour market which may also impact future agricultural activities in the area[9].
**Input data**

Population number at the LAU2 (municipality) level[10].
**Calculation process**<br>$$\frac{\text{Residents age } > 65}{\text{Residents age } 0 - 15}$$
**Standardization**<br>0 = high aging index; 1 = low aging index.

Dependency ratio

**General description**<br>The ratio between the dependent population and the working population per municipality.
**Rationale**<br>This indicator shows the socioeconomic burden on the active population, which must support the non-active population through taxes. It gives insight into structural weaknesses of regions, such as emigration of the economically active population, or their economic attractiveness.
**Input data**<br>Population number at the LAU2 (municipality) level[10].
**Calculation process**<br>$$\frac{\text{Residents age } < 15 \text{ and } > 65}{\text{Residents age } 15 - 65}$$
**Standardization**<br>0 = high dependency ratio; 1 = low dependency ratio.

Population density

**General description**<br>The population density per agricultural area and municipality, (n°/ha).
**Rationale**<br>This indicator gives insight into the rural-urban gradient of each municipality. Dense population clusters generally coincide with urban or metropolitan areas, while lowly populated areas are associated with the countryside. A high population density positively impacts the value of farmland and the availability of labour force, thereby facilitating the continuation of agricultural activity. Very low values on the other hand show a low attractiveness of the region with negative demographic and socioeconomic trends.
**Input data**<br>GISCO population dataset[11], Corine Land Cover[12], EUcropmap[13].
**Calculation process**<br>Calculation of the ratio n° of people/total agricultural area. To calculate the agricultural area per municipality we used the Corine Land Cover classes related to agriculture as a primary source. We used the EUcropmap to obtain information in municipalities for which no agricultural areas were present in the Corine Land Cover.
**Standardization**<br>0 = lower population; 1 = higher population.

Road length

General description
**General description**<br>The total length of the roads per municipality (m).
**Rationale**<br>The availability of transport infrastructure is critical for the adaptive capacity[14]. In the context of viticulture, the available road network is related to the capacity to effectively supply and manage vineyards, for instance when altering varieties or relocating plantations. An increased availability of roads is therefore related to increased adaptive capacity, as many structural changes in vineyards, which may be critical for climate change adaptation, can be performed more effectively.
**Input data**<br>Road shapefile from Open Street Map[15].
**Calculation process**<br>Calculation of the total length of the roads classified as primary, secondary, tertiary, and tracks (up to the 5th grade) in the Open Street Map dataset within a municipality in areas below 1200m of elevation.
**Standardization**<br>0 = lower road length; 1 = higher road length.

Mechanization index

General description
**General description**<br>The value of machinery & equipment per total utilised agricultural area.
**Rationale**<br>A low value indicates regions with low necessity for machinery or very extensive vineyards per farm in which case the depreciations should be easy to manage. High indicator values show machine intensive viticulture in small farm areas. In accord with climate change mitigation policies that aim to reduce the use of fossil fuels in agriculture, a lower dependency on machines relates to a higher adaptive capacity.
**Input data**<br>Statistical data about machinery and equipment and total utilised agricultural area for viticultural farms in Europe (codes in brackets in the calculation process description identify the statistics that have been used to calculate the indicator)[16]
**Calculation process**<br>$\frac{\text{Machinery and equipment (€)}}{\text{Total Utilised Agricultural Area (ha)}}$<br><br>Machinery and equipment (SE455) in € = Tractors, motor cultivators, lorries, vans, cars, major and minor farming equipment.<br>Total Utilised Agricultural Area (SE025) in ha = Total utilised agricultural area of holding. It consists of land in owner occupation, rented land, land in share-cropping (remuneration linked to output from land made available).
**Standardization**<br>0 = high mechanization index; 1 = low mechanization index.

Naturalness

General description
**General description**<br>The natural and semi-natural areas in % of the total area of the municipality.
**Rationale**

Natural and seminatural areas play an important role for viticulture because they provide habitats for natural predators that can support pest and disease control[17]. Because a shift in climatic conditions can change patterns of crop pathogens and pests[18], these areas are of critical importance for adaptation purposes, as they support the resilience of vineyards.
**Input data**<br>Corine Land Cover[12], European DEM[19]
**Calculation process**<br>To calculate the percentage of natural and seminatural areas, we used the Corine Land Cover classes related to forest and seminatural areas, wetlands and water bodies, below 1200m of elevation.
**Standardization**<br>0 = low amount of natural and seminatural areas; 1 = high amount of natural and seminatural areas.

Shift in space

**General description**<br>The potential of a municipality to relocate vineyards to areas with cooler climatic conditions to adapt to increasing temperature, (km^2).
**Rationale**<br>Relocating vineyards to areas with cooler climatic conditions can be an effective adaptation strategy in the context of climate change[7]. For instance, a shift to higher elevations allows to maintain cool climatic conditions during grape ripening under climate change and can thus preserve varietal composition and traditional wine style in a region[20]. The more potential new area is available within the boundaries of a region, the higher its adaptive capacity.
**Input data**<br>Chelsa high resolution monthly average temperature data (present time 1981-2010 and future projection 2071-2100 using the ssp858 scenario and a 5-model ensemble)[5,6], European DEM[19] , Corine Land Cover[12], boundaries of wine regions in Europe[21,22].
**Calculation process**<br>Calculation of present and future Huglin index. All areas that are: too cool for viticulture in the present (Huglin<1200), suitable for viticulture in the future (Huglin>1200) and classified as agricultural or forest areas are considered as potential new areas for viticulture[25–27].
**Standardization**<br>0 = lower capacity to shift in space; 1 = higher capacity to shift in space.

Water availability

**General description**<br>The water available through precipitation in a municipality, (mm).
**Rationale**<br>Artificial irrigation might not be feasible in all areas and can pose significant challenges to the natural water reserves of a region. The available water from precipitation (after accounting for evapotranspiration) is therefore critical to the adaptive capacity of a region. A higher availability of water is related to lower risk for drought and lower dependence on irrigation and may therefore protect regions from negative impacts of climate change.
**Input data**<br>Climate moisture index over the period 1981-2010 from the CHELSA database[5,6], boundaries of wine regions in Europe[21,22].
**Calculation process**

The difference between evapotranspiration and total precipitation.
**Standardization**<br>0 = lower water availability; 1 = higher water availability.

Availability of climatic niches

**General description**<br>The number of climatic niches present in each municipality based on the spatial variability of temperature within the region (°C).
**Rationale**<br>The availability of climatic niches plays an important role for viticulture, since varieties typically have a very narrow range of climatic conditions where they can produce optimum quality[23]. A high spatial variability of climatic conditions within a region thus allows the cultivation of a greater number of varieties and facilitates potential climate change adaptation through relocation of vineyards[24].
**Input data**<br>Chelsa high resolution monthly average temperature data (1981-2010)[5,6], boundaries of wine regions in Europe[21,22].
**Calculation process**<br>Standard deviation of the mean temperature values.
**Standardization**<br>0 = lower availability of climatic niches; 1 = higher availability of climatic niches.

Labour force

**General description**<br>Farm labour force based on the ratio between regular and total labour force.
**Rationale**<br>Regular labour force usually lives in close proximity to the farm where it works. Areas with a higher share of regular labour force from total labour have potentially more access to workers that can be used for specific tasks, e.g., to carry out tailored adaptation strategies.
**Input data**<br>Statistics on labor force in Europe[25], boundaries of wine regions in Europe[21,22].
**Calculation process**<br>$\frac{\text{Regular labour force}}{\text{Total labour force}}$<br>Regular labour force=Labour force that is regularly employed by the farm, and it’s not part of the holding family<br>Total labour force= Total labour force employed by the farm
**Standardization**<br>0 = lower ratio; 1 = higher ratio.

Education level

**General description**<br>The training level of farm managers based on their highest education level.
**Rationale**

A higher education can improve adaptive capacity and the identification of adaptation solutions amongst farmers[26]. Farmers with a higher education level are better prepared to adapt to climate change and adapt their behaviours and consequently have a higher adaptive capacity.

Input data

Statistics on training of farm managers in Europe[27], boundaries of wine regions in Europe[21,22].

Calculation process

$$\frac{\frac{\text{Farmers with full education} * 2}{\text{Number of total farmers}} + \frac{\text{Farmers with basic education}}{\text{Number of total farmers}}}{2}$$

Farmers with full education = Farmers with full agricultural training

Farmers with basic education = Farmers with basic agricultural training

Number of total farmers = Total number of farmers in the region

Standardization

0 = lower level of training; 1 = higher level of training.

Research accessibility

General description

The distance of each LAU2 polygon included in a winegrowing region to the next major research centre on wine and vine in Europe, (m).

Rationale

Research and technology are important to find new solutions for climate change adaptation[28]. A lower distance to major research centres facilitates the transmission of new knowledge and innovative solutions and therefore improves adaptive capacity.

Input data

Location of the research centers on wine and vine, boundaries of LAU2 polygons[11], OSM European road network[15], boundaries of wine regions in Europe[21,22].

Calculation process

Calculation of the linear distance along the roads from the centroid of each LAU2 polygon included in a winegrowing region to the nearest research centre on vine and wine. The research centres were selected based on a search in the Scopus database: we searched papers published in the last 5 years on the topic of viticulture and spatialized the affiliation of the first authors in the list.

Standardization

0 = low distance to research centres; 1 = high distance to research centres.

Debt ratio

General description

The liability percentage of the total assets of farms specialized in viticulture. This indicator shows how much of the farm capital is owned by the farmer and how much is borrowed capital (%).

Rationale

Financial readiness is important for farms in the context of climate change, as they might need to adopt new innovative solutions and technologies to cope with the negative impacts[26]. Lower values of this indicator show regions where farms are less indebted and therefore can acquire additional capital and pay back already borrowed capital more easily in case of needed investments, e.g., to develop strategies for climate change adaptation.

Input data

Statistical data about liabilities and total assets for viticultural farms in Europe (codes in brackets in the calculation process description identify the statistics that have been used to calculate the indicator)[16].
**Calculation process**<br>$$\frac{\text{Total liabilities}}{\text{Total assets closing valuation}}$$<br>Total liabilities (SE485) = Value at closing valuation of total of (long-, medium- or short-term) loans still to be repaid.<br>Total assets closing valuation (SE436) = Fixed assets + current assets.
**Standardization**<br>0 = higher ratio; 1 = lower ratio.

Return on assets

**General description**<br>The adjusted net income of farms specialized in viticulture compared to their total assets (%).
**Rationale**<br>This indicator is a measure of fiscal health of the farm by showing the real farm profitability. Farms specialised in viticulture are asset intensive so return on assets of more than 3% shows already success. A high value indicates farms that are economically successful and therefore have a higher adaptive capacity to climate change, as it is easier to write off investments or pay for additional labour and still be profitable.
**Input data**<br>Statistical data about the return on assets for viticultural farms in Europe (codes in brackets in the calculation process description identify the statistics that have been used to calculate the indicator)[16].
**Calculation process**<br>$$\frac{(\text{Farm Net Income} - (\text{Unpaid labour input} * (\text{Wages paid}/\text{Paid labour input})))}{\text{Total assets closing valuation}}$$<br>Farm net income (SE420) in € = Remuneration to fixed factors of production of the family and remuneration to the entrepreneur's risks in the accounting year.<br>Unpaid labour input (SE015) = Refers to unpaid labour expressed in annual work units (AWU) → labour input by the farmer or the farmers family.<br>Wages paid (SE370) in € = Wages and social security charges of wage earners.<br>Paid labour input (SE020) = Refers to paid labour expressed in AWU.<br>Total assets closing valuation (SE436) in € = Fixed assets plus current assets of the farm.
**Standardization**<br>0 = lower ratio; 1 = higher ratio.

Subsidy dependence

**General description**<br>Dependency of farms in the viticultural sector on subsidies (%).
**Rationale**

Farms with a low subsidy dependency are economically more viable on their own. The higher the ratio the less profitable are farms if subsidies are discontinued.
**Input data**<br>Statistical data about the return on percentage of subsidies and net income for viticultural farms in Europe (codes in brackets in the calculation process description identify the statistics that have been used to calculate the indicator)[16].
Calculation process<br>$$\frac{\text{Total subsidies} \; - \; \text{excluding on investments}}{\text{Farm Net Value Added}}$$<br>Total subsidies - excluding on investments (SE605) = Subsidies on current operations linked to production, in €.<br>Farm Net Value Added (SE415) = Remuneration to the fixed factors of production (work, land and capital), whether they be external or family factors.
**Standardization**<br>0 = higher ratio; 1 = lower ratio.

Supplementary Note 2: Validation of the bioregional climate range

To validate the bioregional climate ranges that were used to calculate the climate change sensitivity, we compared our results with two previous studies: i) climate ranges for several international varieties derived from global high-quality wine regions[29] and ii) climate ranges for several national varieties from a study focusing on wine regions in Portugal[30]. In both cases, we selected the 10 varieties with the largest cultivation area according to ref[31] for the comparison to our approach. To be able to compare the climatic ranges, we used the approach as presented in the main text but used the same bioclimatic index as in the corresponding publication: growing degree days (GDD) with a base temperature of 10°C for ref[30] and the average temperature during the growing season from April to October for ref[29]. Moreover, the regions were not grouped into climatic classes, because no such subdivision was present in the original publications.

Even though the ranges in both studies were identified based on detailed local/regional climatic and geographic information for individual varieties, we observed a very high correspondence of both studies to our bioregional climate range which is based on much coarser European-scale data (Supplementary Figure 13). This indicates that our approach can capture intra-varietal differences and accurately estimates climatic ranges across several different varieties. However, there are also some clear limitations. Especially for varieties with very narrow climatic ranges our approach tends to over- or underestimate the climatic range. There are also some international varieties for which our approach over- or underestimates the range of climatic conditions compared to previously published ranges (e.g., Syrah or Tempranillo). Differences between the ranges in our approach and those from previous studies may be related to i) the inclusion of regions in our approach that were not considered in the original publications or ii) a mismatch between average growing conditions within a PDO, as used in our approach, and actual growing conditions at the planting locations of the respective variety. Despite these limitations, the high correlation for both local Portuguese varieties and international varieties shows that our approach can provide a reasonable estimation for the typical climatic range of different vine varieties

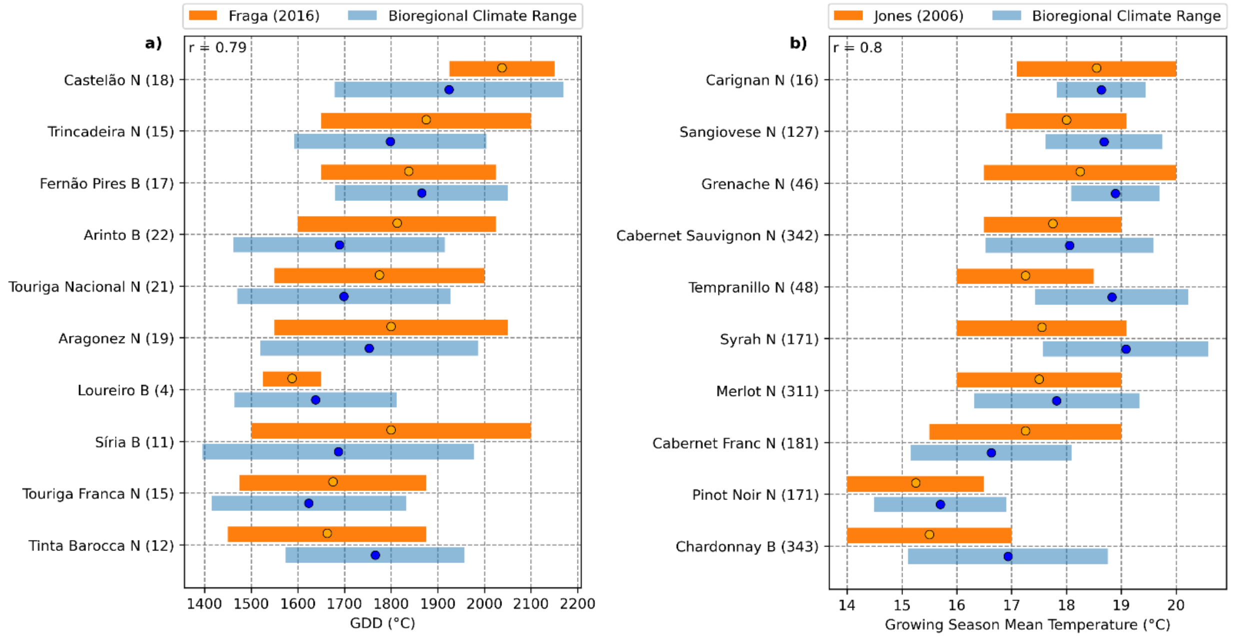

Supplementary Figure 14: Comparison of climatic ranges for several varieties from two peer-reviewed studies (orange bars) with the present approach (blue bars). The points indicate the median of the climatic range and the coloured bars the entire range of conditions under which the variety is cultivated In contrast to the approach presented in the main text, the regions were not grouped into climatic classes for this comparison, because no such subdivision was present in the original publications, and the calculation was based on the same bioclimatic indices as in the corresponding publication. To derive the bioregional climate range (blue bar) in a) we only considered PDO regions in Portugal (n=30) while for b) all PDOs (n=1085) in Europe were used. The number of regions that were considered to determine the range for each variety is indicated in brackets after the variety name. r = pearson correlation

Supplementary References

1. Fraga, H. *et al.* Very high resolution bioclimatic zoning of Portuguese wine regions: present and future scenarios. *Reg Environ Change* **14**, 295–306 (2014).

2. Fraga, H., Malheiro, A. C., Moutinho-Pereira, J. & Santos, J. A. Future scenarios for viticultural zoning in Europe: Ensemble projections and uncertainties. *Int J Biometeorol* **57**, 909–925 (2013).

3. Fraga, H. *et al.* Climatic suitability of Portuguese grapevine varieties and climate change adaptation. *Int J Climatol* **36**, 1–12 (2016).

4. Santillán, D., Iglesias, A., Jeunesse, I. L., Garrote, L. & Sotes, V. Vineyards in transition: A global assessment of the adaptation needs of grape producing regions under climate change. *Sci Total Environ* **657**, 839–852 (2019).

5. Karger, D. N. *et al.* Climatologies at high resolution for the earth's land surface areas. *Sci Data* **4**, 170122 (2017).

6. Karger, D. N. *et al.* Data from: Climatologies at high resolution for the earth's land surface areas. *EnviDat* (2018) doi:10.16904/envidat.228.v2.1.

7. Santos, J. A. *et al.* A Review of the Potential Climate Change Impacts and Adaptation Options for European Viticulture. *Appl Sci* **10**, 3092 (2020).

8. Bois, B., Zito, S. & Calonnec, A. Climate vs grapevine pests and diseases worldwide: the first results of a global survey. *Oeno One* **51**, 133–139 (2017).

9. Borsdorf, A., Tasser, E. I. & Tappeiner, U. *Alpenatlas: Society - Economy - Environment*. (Spektrum Akademischer Verlag, 2008).

10. Eurostat. Eurostat Census Hub. https://ec.europa.eu/CensusHub2 (2022).

11. Eurostat. GISCO LAU2 dataset. https://ec.europa.eu/eurostat/web/gisco/geodata.

12. European Union, Copernicus Land Monitoring Service & European Environment Agency. *CORINE Land Cover 2018*. https://land.copernicus.eu/en/products/corine-land-cover/clc2018 (2020).

13. d'Andrimont, R. *et al.* From parcel to continental scale – A first European crop type map based on Sentinel-1 and LUCAS Copernicus in-situ observations. *Remote Sens Environ* **266**, 112708 (2021).

14. Pörtner, H.-O. *et al. Climate Change 2022: Impacts, Adaptation and Vulnerability*. (Cambridge University Press, Cambridge, 2022). doi:10.1017/9781009325844.002.

15. OpenStreetMap Contributors. *OpenStreetMap Country Shapefiles*. https://download.geofabrik.de/.

16. European Union. Farm accountancy data network (FADN) Public Database. https://agridata.ec.europa.eu/extensions/DashboardFarmEconomyFocus/DashboardFarmEconomyFocus.html (2022).

17. Winkler, K. J., Viers, J. H. & Nicholas, K. A. Assessing Ecosystem Services and Multifunctionality for Vineyard Systems. *Frontiers Environ Sci* **5**, 15 (2017).

18. Caffarra, A., Rinaldi, M., Eccel, E., Rossi, V. & Pertot, I. Modelling the impact of climate change on the interaction between grapevine and its pests and pathogens: European grapevine moth and powdery mildew. *Agric Ecosyst Environ* **148**, 89–101 (2012).

19. European Union & Copernicus Land Monitoring Service. European Digital Elevation Model (EU-DEM), Version 1.1. https://land.copernicus.eu/pan-european/satellite-derived-products/eu-dem/eu-dem-v1.1/ (2016).

20. Egarter Vigl, L. *et al.* Upward shifts in elevation – a winning strategy for mountain viticulture in the context of climate change? in *E3S Web of Conferences* vol. 50 02006 (2018).

21. Candiago, S., Tscholl, S., Bassani, L., Fraga, H. & Egarter Vigl, L. A geospatial inventory of regulatory information for wine protected designations of origin in Europe. *Sci Data* **9**, 394 (2022).

22. Candiago, S., Tscholl, S., Bassani, L., Fraga, H. & Egarter Vigl, L. A geospatial inventory of regulatory information for wine Protected Designations of Origin in Europe. *Figshare* (2022) doi:10.6084/m9.figshare.c.5877659.v1.

23. Parker, A. K. *et al.* Temperature-based grapevine sugar ripeness modelling for a wide range of Vitis vinifera L. cultivars. *Agr Forest Meteorol* **285–286**, 107902 (2020).

24. Tscholl, S., Tasser, E., Ulrike, T. & Egarter Vigl, L. Coupling solar radiation and cloud cover data for enhanced temperature predictions over topographically complex mountain terrain. *Int J Climatol* **42**, 4684–4699 (2021).

25. Eurostat. Eurostat data explorer: Labour force main indicators. (2021) doi:10.2908/ef_lf_size.

26. Williges, K., Mechler, R., Bowyer, P. & Balkovic, J. Towards an assessment of adaptive capacity of the European agricultural sector to droughts. *Clim Serv* **7**, 47–63 (2017).

27. Eurostat. Eurostat Data Browser: Training of Farm Managers. doi:10.2908/ef_mp_training.

28. Greiving, S. *et al. ESPON Climate: Climate Change and Territorial Effects on Regions and Local Economies*. https://www.espon.eu/sites/default/files/attachments/Final%20Report%20Main%20Report.pdf (2011).

29. Jones, G. V. Climate and terroir: impacts of climate variability and change on wine. *Fine Wine and Terroir - The Geoscience Perspective* 247 (2006).

30. Fraga, H. *et al.* Climatic suitability of Portuguese grapevine varieties and climate change adaptation. *Int J Climatol* **36**, 1–12 (2016).

31. Anderson, K. & Nelgen, S. *Database of Regional, National and Global Winegrape Bearing Areas by Variety, 1960 to 2016*. (University of Adelaide, South Australia, 2020).
