# Supplementary material for: Climate resilience of European wine regions
Source: Nat Commun. 2024 Jul 24;15:6254. doi: 10.1038/s41467-024-50549-w (PMC11269675; doi:10.1038/s41467-024-50549-w)
Supplement: Supplementary file 3 — Reporting Summary [file 41467_2024_50549_MOESM3_ESM.pdf]

nature portfolio

Corresponding author(s): Simon Tscholl
Last updated by author(s): Jun 11, 2024

Reporting Summary

Nature Portfolio wishes to improve the reproducibility of the work that we publish. This form provides structure for consistency and transparency in reporting. For further information on Nature Portfolio policies, see our Editorial Policies and the Editorial Policy Checklist.

Statistics

For all statistical analyses, confirm that the following items are present in the figure legend, table legend, main text, or Methods section.

n/a	Confirmed	
☐	☒	The exact sample size (*n*) for each experimental group/condition, given as a discrete number and unit of measurement
☒	☐	A statement on whether measurements were taken from distinct samples or whether the same sample was measured repeatedly
☒	☐	The statistical test(s) used AND whether they are one- or two-sided<br>*Only common tests should be described solely by name; describe more complex techniques in the Methods section.*
☒	☐	A description of all covariates tested
☒	☐	A description of any assumptions or corrections, such as tests of normality and adjustment for multiple comparisons
☐	☒	A full description of the statistical parameters including central tendency (e.g. means) or other basic estimates (e.g. regression coefficient) AND variation (e.g. standard deviation) or associated estimates of uncertainty (e.g. confidence intervals)
☒	☐	For null hypothesis testing, the test statistic (e.g. F, t, r) with confidence intervals, effect sizes, degrees of freedom and P value noted<br>*Give P values as exact values whenever suitable.*
☒	☐	For Bayesian analysis, information on the choice of priors and Markov chain Monte Carlo settings
☒	☐	For hierarchical and complex designs, identification of the appropriate level for tests and full reporting of outcomes
☐	☒	Estimates of effect sizes (e.g. Cohen's *d*, Pearson's *r*), indicating how they were calculated

Our web collection on statistics for biologists contains articles on many of the points above.

Software and code

Policy information about availability of computer code

Data collection	We used Microsoft Excel (version 2405) and GIS Software (ArcGIS Pro 3.2.2 & QGIS 3.34) to collect the relevant data
Data analysis	To analyze the data in this study we used Microsoft Excel (version 2405) and GIS Software (ArcGIS Pro 3.2.2 & QGIS 3.34)

For manuscripts utilizing custom algorithms or software that are central to the research but not yet described in published literature, software must be made available to editors and reviewers. We strongly encourage code deposition in a community repository (e.g. GitHub). See the Nature Portfolio guidelines for submitting code & software for further information.

Data

Policy information about availability of data

All manuscripts must include a data availability statement. This statement should provide the following information, where applicable:

- Accession codes, unique identifiers, or web links for publicly available datasets
- A description of any restrictions on data availability
- For clinical datasets or third party data, please ensure that the statement adheres to our policy

The dataset containing the primary and additional varieties cultivated in each PDO generated in this study has been deposited in the Zenodo database under accession code https://doi.org/10.5281/zenodo.7257126. The dataset containing the exposure, sensitivity and adaptive capacity indicators generated in this study has also been deposited in the Zenodo database under accession code https://zenodo.org/records/10410972. The sources for the data underlying the individual indicators are provided in the supplementary materials file alongside the description of each indicator (Supplementary Note 1).

Research involving human participants, their data, or biological material

Policy information about studies with human participants or human data. See also policy information about sex, gender (identity/presentation), and sexual orientation and race, ethnicity and racism.

Reporting on sex and gender	This information has not been collected
Reporting on race, ethnicity, or other socially relevant groupings	See above
Population characteristics	See above
Recruitment	See above
Ethics oversight	See above

Note that full information on the approval of the study protocol must also be provided in the manuscript.

Field-specific reporting

Please select the one below that is the best fit for your research. If you are not sure, read the appropriate sections before making your selection.

☐ Life sciences ☐ Behavioural & social sciences ☒ Ecological, evolutionary & environmental sciences

For a reference copy of the document with all sections, see nature.com/documents/nr-reporting-summary-flat.pdf

Ecological, evolutionary & environmental sciences study design

All studies must disclose on these points even when the disclosure is negative.

Study description	We analyzed the climate change vulnerability of 1085 European PDO regions by dividing them into homogeneous groups based on their exposure, sensitivity and adaptive capacity.
Research sample	We used all the wine PDO regions within the European Union for which we had sufficient data which is a total of 1085 regions. We used a database that we created and that is freely available online, related desciptions and link for download can be found here: https://doi.org/10.1038/s41597-022-01513-0.
Sampling strategy	Since our study aim was to understand climate change impacts on high-quality wine production in Europe, we focused on PDO regions because they have the strongest link to a defined production area and its climate. To have a comprehensive overview, we included all available PDO regions where we had sufficient data in our study.
Data collection	The data was collected from different sources: statistical databases, regulatory documents and geo-data repositories. The data collection was carried out by S.T., S.C. and T.M.<br>The sources for the data underlying the individual indicators are provided in the supplementary materials file alongside the description of each indicator (Supplementary Note 1).
Timing and spatial scale	The data related to this publication was collected and processed during the period January 2022 - August 2022. The spatial scale strongly depends on the indicator; some were available at the national scale while others were available at the municipality level. For the aim of this study all the indicators were standardized at the scale of each PDO.
Data exclusions	Regions for which we did not have sufficient data were excluded.
Reproducibility	The main calculations were performed in Microsoft Excel (version 2405) and GIS Software (ArcGIS Pro 3.2.2 & QGIS 3.34).
Randomization	Randomization was not relevant for our study.
Blinding	Blinding was not relevant for our study.

Did the study involve field work? ☐ Yes ☒ No

Reporting for specific materials, systems and methods

We require information from authors about some types of materials, experimental systems and methods used in many studies. Here, indicate whether each material, system or method listed is relevant to your study. If you are not sure if a list item applies to your research, read the appropriate section before selecting a response.

Materials & experimental systems

n/a	Involved in the study
☒	☐ Antibodies
☒	☐ Eukaryotic cell lines
☒	☐ Palaeontology and archaeology
☒	☐ Animals and other organisms
☒	☐ Clinical data
☒	☐ Dual use research of concern
☒	☐ Plants

Methods

n/a	Involved in the study
☒	☐ ChIP-seq
☒	☐ Flow cytometry
☒	☐ MRI-based neuroimaging
